# The Heart Remembers: A Case of Cardiac Memory

**DOI:** 10.7759/cureus.42106

**Published:** 2023-07-19

**Authors:** Riddhima Naik, Harshith Thyagaturu, Maan Awad, Edward Bischof

**Affiliations:** 1 Internal Medicine, Bassett Healthcare Network, Cooperstown, USA; 2 Cardiology, West Virginia University School of Medicine, Morgantown, USA; 3 Internal Medicine, West Virginia University School of Medicine, Morgantown, USA

**Keywords:** acute coronary syndrome, pacemaker, syncope, ventricular paced rhythm, cardiac memory

## Abstract

An 80-year-old male with a history of atrial fibrillation and a single-chamber ventricular pacemaker presented to the hospital for an elective colonoscopy. He experienced a transient episode of unresponsiveness with seizure-like activity before the procedure. This prompted him to get an EKG showing deep T-wave inversions (TWIs) in the precordial leads on a background of paced beats. Such findings were concerning for an acute and potentially life-threatening process such as myocardial infarction (MI) or intracranial insult. After ruling out any severe conditions, the EKG findings were attributed to cardiac memory, an underdiagnosed cause of deep TWIs in patients with a pacemaker.

## Introduction

Cardiac memory (CM) is a phenomenon characterized by changes in cardiac electrical conduction that persist after a period of altered pacing. It is thought to occur as a result of changes in the expression and distribution of ion channels in the myocardium, which can lead to a prolonged action potential duration and T-wave inversion (TWI) on the electrocardiogram (ECG) [1,2]. The characteristic pattern is a reversal of T-wave polarity in leads with abnormal repolarization during the memory phase. These ECG changes may be transient or persistent, depending on the underlying condition and the duration of the stimulus. Cardiac memory has been documented in various clinical scenarios, such as pacing changes, atrial fibrillation, and ventricular arrhythmias. While the underlying mechanisms of cardiac memory remain incompletely understood, it is considered an under-recognized and under-diagnosed phenomenon [2]. Although cardiac memory is generally considered a benign phenomenon that can occur in hearts without any evidence of hemodynamic instability, structural abnormalities, or ischemic compromise, the resulting TWI can mimic the ECG findings observed in pathological conditions like myocardial ischemia or infarction. Awareness of this condition can help improve clinical decision-making and patient outcomes.

## Case presentation

**Figure 1:**
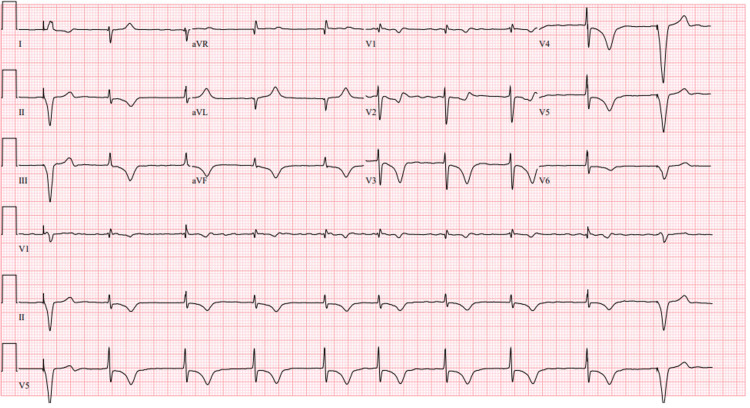
Deep TWI noted in the precordial leads (V3, V4, and V5), representing the cardiac memory phenomenon. TWI: T-wave inversion.

**Figure 2:**
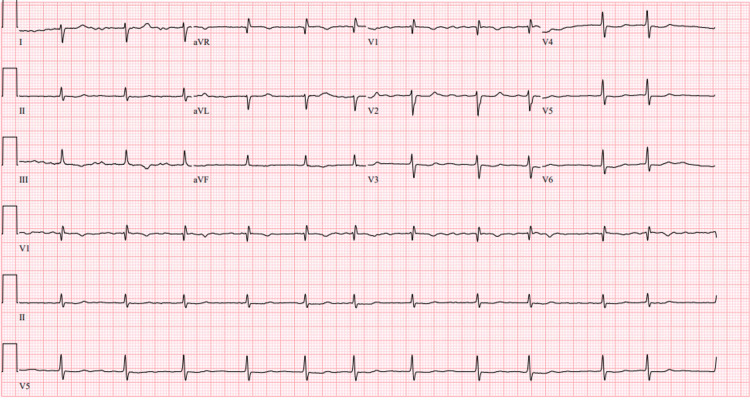
Baseline EKG of the patient in atrial fibrillation before pacemaker insertion.

**Figure 3:**
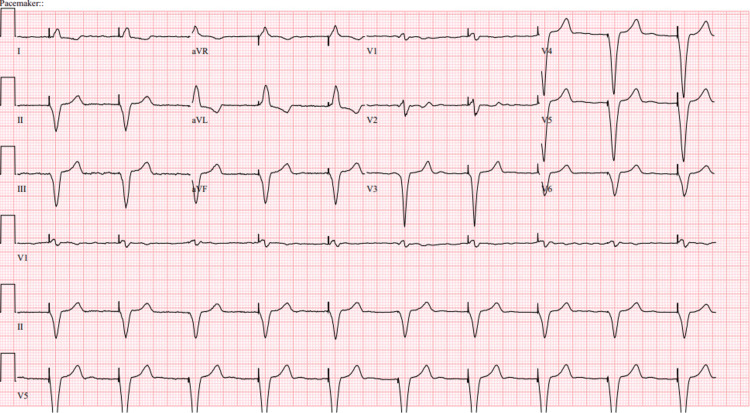
Baseline EKG of the patient shortly after pacemaker insertion. Of note, no T-wave changes were present.

An 80-year-old male with a medical history of atrial fibrillation, a single-chamber ventricular pacemaker, and severe pulmonary hypertension was admitted to the hospital for an elective colonoscopy. While in the preoperative area, the patient experienced a transient episode of unresponsiveness with seizure-like activity, prompting him to obtain an ECG. The patient reported having had similar attacks in the past, with a previous workup revealing no underlying organic cause. He denied any chest pain, dyspnea, orthopnea, dizziness, or lightheadedness and quickly fell back to baseline. Upon examination, the patient had a blood pressure of 106/65 mmHg, a heart rate of 60 beats/min, and a saturation of 99% in room air. There was prominent jugular venous distention, a regular heart rate and rhythm, a 2/6 systolic ejection murmur at the right upper sternal border, and clear lungs. The patient did not exhibit any focal neurological deficits, and his neurological exam was grossly intact. An admission ECG revealed rate-controlled atrial fibrillation with deep TWIs, most prominent in precordial leads (V3, V4, and V5), and paced beats at the beginning and end of the ECG strip (Figure 1). The patient's baseline ECG from when he was in atrial fibrillation and when he was in paced rhythm is noted in Figures 2, 3. Laboratory and imaging findings were unremarkable, with normal troponin levels and a head CT scan without contrast. The patient's pacemaker was interrogated, revealing that he was primarily ventricular paced (86%), which was markedly increased from a year ago (26%).

Given this patient’s presentation with transient unresponsiveness and seizure-like activity, there was a concern for other causes of isolated prominent TWIs, including cerebral T waves, which could be seen in patients with cerebral hemorrhage and other conditions associated with increased intracranial pressure. This was ruled out with a non-focal neurological exam, a history of prior similar episodes, and a negative head CT scan. An acute myocardial infarction (MI), manifesting as Wellens syndrome, was also of concern. This was less likely given the normal troponin levels and unremarkable transthoracic echocardiogram. The episode lasted a few seconds, and the patient returned to baseline. After excluding potential life-threatening conditions, the TWI noted on the patient's ECG was attributed to cardiac memory.

## Discussion

The ECG findings in Figure 1 of this case were attributed to cardiac memory (CM), an underdiagnosed etiology of deep T-wave inversions (TWIs) in patients with a pacemaker [3]. These deep TWIs usually occur after prolonged pacing and are thought to result from T waves in sinus rhythm "remembering" the direction of the QRS complex during pacing [3]. The abnormal ventricular activation pattern during pacing leads to electrical remodeling of the myocardial cells, which manifests as changes in the T wave vector and polarity during conversion to the native rhythm [4]. Abnormal ventricular activation due to ventricular pre-excitation, intermittent left bundle branch block, wide QRS rhythms, and ventricular tachycardia can also cause CM [5]. The duration of memory T waves is proportional to the pacing duration, a phenomenon also known as "accumulation" [6,7]. In the presented case, memory T waves were detected only after a significant increase in the pacing duration from a year ago (from 26% to 86% V-paced).

In addition to CM, other potential causes of isolated prominent TWIs were considered in this patient, including cerebral T waves and Wellens syndrome. Cerebral T waves are deep TWIs seen after intracranial hemorrhage or raised intracranial pressure, and they can present with additional ECG changes such as Osborne waves, prolonged QT intervals, and various dysrhythmias [7]. Another concern was acute MI, manifested as Wellens syndrome, which requires prompt intervention due to transient occlusion of the proximal left anterior descending artery. Troponin levels can be negative during an acute coronary event, and further criteria have been developed to assist in ruling out MI. A retrospective study has described ECG criteria to differentiate CM from ischemia based on the T wave axis and polarity, with reported sensitivity and specificity of 92% and 100%, respectively, for CM [8]. The criteria include: (1) positive T wave in aVL with positive or isoelectric T wave in lead I, and (2) amplitude of precordial TWI > inferior lead TWI in lead III. Both criteria were present in this case, suggesting the absence of ischemia. Acute pericarditis is another potential cause of deep TWIs, commonly demonstrating diffuse ST segment elevation and PR interval depression [9]. Hypokalemia can also produce TWI, but the T waves are often more flattened and accompanied by prominent U waves, prolonged PR interval, and diffuse ST depression [10]. Cardiac memory does not require any specific intervention, but it is essential to rule out life-threatening causes of deep TWI, such as acute MI and cerebral events.

## Conclusions

Cardiac memory is an underdiagnosed cause of deep TWI in patients with a pacemaker. It is caused by electrical remodeling of the myocardial cells due to abnormal ventricular activation during pacing, leading to changes in the T-wave vector and polarity during conversion to the native rhythm. The duration of memory T waves appears to be proportional to the pacing duration. We describe a case of an 80-year-old male who was found to have deep TWIs on the ECG after sustaining a transient episode of unresponsiveness. The ECG changes were quite concerning on the initial assessment, representing a marked change from his baseline ECG. After ruling out life-threatening and other causes of such ECG changes, a diagnosis of cardiac memory was made, supported by the increased proportion of paced rhythm. Although CM does not require any specific intervention, ruling out life-threatening causes of deep TWI, such as acute MI and cerebral events, is essential. Further studies are needed to improve our understanding of CM and its long-term clinical implications.
